# Simple Method for Sub-Diffraction Resolution Imaging of Cellular Structures on Standard Confocal Microscopes by Three-Photon Absorption of Quantum Dots

**DOI:** 10.1371/journal.pone.0064023

**Published:** 2013-05-21

**Authors:** Anje Sporbert, Zoltan Cseresnyes, Meike Heidbreder, Petra Domaing, Stefan Hauser, Barbara Kaltschmidt, Christian Kaltschmidt, Mike Heilemann, Darius Widera

**Affiliations:** 1 Confocal and 2-Photon Microscopy Core Facility, Max-Delbrueck-Center for Molecular Medicine Berlin, Berlin, Germany; 2 Department of Biotechnology and Biophysics, Julius-Maximilians-Universität, Am Hubland, Würzburg, Germany; 3 Molecular Neurobiology, University of Bielefeld, Bielefeld, Germany; 4 LiMiTec, Light Microscopy Technology Platform, University of Bielefeld, Bielefeld, Germany; 5 Cell Biology, University of Bielefeld, Bielefeld, Germany; 6 Physical and Theoretical Chemistry, Goethe-University Frankfurt, Frankfurt/Main, Germany; Argonne National Laboratory, United States of America

## Abstract

This study describes a simple technique that improves a recently developed 3D sub-diffraction imaging method based on three-photon absorption of commercially available quantum dots. The method combines imaging of biological samples via tri-exciton generation in quantum dots with deconvolution and spectral multiplexing, resulting in a novel approach for multi-color imaging of even thick biological samples at a 1.4 to 1.9-fold better spatial resolution. This approach is realized on a conventional confocal microscope equipped with standard continuous-wave lasers. We demonstrate the potential of multi-color tri-exciton imaging of quantum dots combined with deconvolution on viral vesicles in lentivirally transduced cells as well as intermediate filaments in three-dimensional clusters of mouse-derived neural stem cells (neurospheres) and dense microtubuli arrays in myotubes formed by stacks of differentiated C2C12 myoblasts.

## Introduction

**Figure 1:**
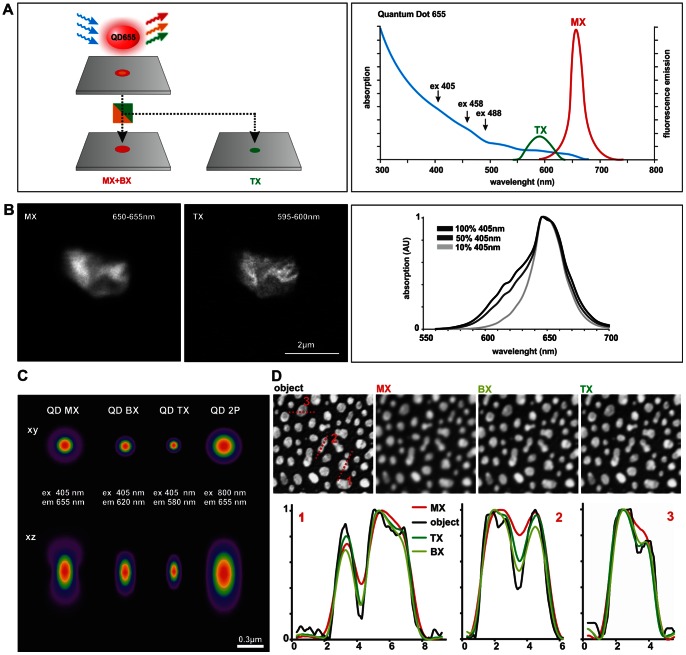
The principle of quantum dot tri-exciton imaging (QDTI). (A) QDTI utilizes the ability of quantum dots QD655 to successively absorb three photons, which is followed by blue-shifted emission arising from higher-order excitonic states. Tri-excitonic (TX) states are centered at 590 nm and can be spectrally separated from mono- (MX) and bi- (BX) excitonic states (Spectra redrawn based on Invitrogen data). (B) Aggregates of QD655 labeled antibodies were imaged with a spectral scan from 550 to 700 nm with a band width of 5 nm. The reduced dimensions of the point spread function (PSF) leads to a resolution enhancement, which is clearly seen as improved structural details in the TX image obtained in the 595–600 nm range. The relative and absolute amount of BX and TX states generated in QD655 by 405 nm depends on the intensity of the excitation laser applied as shown by the increased formation of a shoulder around 610 nm. Intensities of 10% and 50% at 405 nm are in the range of typical laser power used for conventional QD and QDTX images, respectively. C) The effect of different theoretical PSF models on image formation. Lateral and axial views of the theoretical PSF calculated for mono-, bi- and triexciton absorption of QD655, assuming excitation at 405 nm and emission at 655 nm for normal QD imaging (MX), 620 nm emission for 2P (BX) and 580 nm for 3P absorption (TX). For comparison, 2P absorption with excitation at 800 nm and emission at 655 nm are included. D) These theoretical PSFs were applied to convolve an example image from ImageJ, named “blobs”, to simulate the effect of the various PSF models on revealing and hiding structural details of real biological structures. Line profiles show the effect of the PSF models for MX, BX, TX on the separation of nearby structures (line profile 1and 2) and on substructures (line profile 1 and 3).

The observation of cellular structures at the molecular level is hampered by the resolution limit in light microscopy. Structures that are smaller than about 200 nm cannot be discerned with conventional imaging techniques. To circumvent this barrier, a number of new microscopy techniques have recently been developed, improving the spatial resolution to the near-molecular scale [1], [2]. However, the increase in spatial resolution comes at the cost of either more complex microscopic arrangement, the necessity of mathematical post-processing of images, the need for high irradiation powers, or the requirement of very unique fluorescent probes. Recently, we introduced a novel microscopy method that enables imaging of cellular structures with an optical resolution beyond the diffraction limit on the basis of a novel concept that is very different from all other super-resolution techniques described so far [3]. This approach uses commercially available quantum dots (QD) and a conventional confocal microscope with continuous wave (CW) lasers. Due to their spectral flexibility in excitation, high fluorescence brightness and photo-stability, commercially available QD655 are attractive probes for fluorescence imaging. In addition to their applicability in conventional fluorescence microscopy, these cadmium-selenium (CdSe) QDs allow the generation of higher excitonic states by CW irradiation [4] without the need for pulsed multi-photon excitation sources. The underlying mechanism is the subsequent absorption of three photons, which leads to the generation of a tri-excitonic state, a common photophysical effect in quantum dots [5]. Unlike other excitonic states, the tri-exciton state is depopulated by the emission of a photon with higher energy, and thus can be spectrally separated from mono- (MX) and bi-excitonic (BX) emission [4] (Figure 1A). This approach is different from common two- or multi-photon microscopy, where the population of a very short lived and virtual state requires pulsed laser sources at a doubled wavelength and does not lead to an increase in spatial resolution. Quantum dot tri-exciton imaging (QDTI) narrows the excitation point spread function (PSF, Figure 1C) and leads to an experimentally observed approximately up to 1.7-fold increase in spatial resolution. This approach can be used to resolve any kind of cellular structure, e.g. the cytoskeleton [3] or single membrane receptors [6], even in live cells [3]. Here, we extended this concept by developing an appropriate strategy for deconvolution of 3D quantum dot tri-exciton images to further improve the image contrast. In addition, careful selection of organic fluorophores and corresponding excitation wavelengths allows combining QDTI with conventional multi-color confocal imaging of commonly used green and far-red organic fluorophores, as well as nuclear stains. This substantially extends the applicability of QDTI and allows visualization of cellular structures with sub-diffraction resolution. We performed standard immunocytochemical stainings of a wide range of cellular structures in different mammalian cell types using QD655 coupled to secondary antibodies to show the potential of QDTI.

## Materials and Methods

### Ethics Statement

Prior to tissue isolation, mice were kept under specific pathogen free conditions as defined by the Federation European Laboratory Animal Science Association (FELASA) in the central animal facility of Bielefeld University. This study was carried out in strict accordance with the regulations of the governmental animal and care use committee, LANUV of the state North Rhine-Westphalia (Düsseldorf, Germany). All animal tissue isolation-procedures were approved by the Ethical Committee LANUV of the state North Rhine-Westphalia (Düsseldorf, Germany). All efforts were made to minimize animal suffering and the number of sacrificed animals.

### Isolation and Cultivation of Mouse Hippocampal Neural Stem Cells (hNSCs)

Young adult C57/BL6 mice (8–12 weeks) were euthanized by cervical dislocation followed by immediate removal of whole brains. Hippocampi were dissected and transferred into ice-cold 1×Hank’s Buffered Salt Solution (HBSS; Life Technologies/Gibco, Darmstadt, Germany) supplemented with 15 mM HEPES (PAA, Pasching, Austria), D-Glucose (5.44 mg/ml; Sigma-Aldrich, Munich, Germany), Amphotericin B (1∶100; PAA), and Penicillin/Streptomycin (1∶100; PAA). Further stem cell isolation procedure from the dissected tissue was performed as described in [7]. The resulting cell suspension was plated on poly-D-lysin/laminin coated dishes (for coating protocol see [8] in serum-free DMEM/F12 (Biochrom AG, Berlin, Germany) supplemented with 20 ng/ml EGF (R&D Systems, Wiesbaden, Germany), 40 ng/ml FGF-2 (labmade), 3×NS21/B27 supplement [9] and heparin (0.5 U/ml, Sigma-Aldrich) and subsequently cultivated at 37°C, 5% CO_2_ and 5% O_2_ until confluency of ∼80%. Thereafter hNSCs were transferred into low-adhesion T25 tissue cell culture flasks (Greiner Bio One, Frickenhausen, Germany) leading to the formation of self-adherent, free-floating neurospheres. Prior to fixation and immunocytochemical stainings against nestin, neurospheres were harvested in FGF-2 and EGF-free DMEM/F12 containing 10% fetal calf serum (FCS; Sigma-Aldrich, Munich, Germany) and allowed to settle on coverslips placed in 12-well tissue culture test plates (TPP, Trasadingen, Suisse) for 2h at 37°C, 5% CO_2_ and 5% O_2_ to induce attachment, spontaneous differentiation and the resulting depolymerization of nestin filaments.

### Cell Culture of HEK293FT, C2C12 Myoblasts, Myotube Formation and Lentiviral Transduction

HEK293FT cells (Life Technologies/Invitrogen) were cultivated in high glucose DMEM (PAA, Pasching, Austria) supplemented with 10% FCS and Penicillin/Streptomycin (1∶100; PAA). Lentivirus production was performed by co-transfection of 293FT cells using calcium-phosphate precipitation. One day before transfection, 1×10^7^ cells were plated on a 15 cm dish. The next day cells were transfected with FUG-W transfer plasmid, Δ8.91, VSV-G helper plasmids [10] and LacZ-plasmid [11]. For immunocytochemical stainings against lentiviral p24, the whole medium was changed 8 h after transduction and the infected cells were further cultivated for 24 h. Mouse C2C12 myoblast cells (ATCC® CRL 1772 strain C3H)) were grown in DMEM (PAA) supplemented with 10% FCS and Penicillin/Streptomycin (1∶100; PAA). Fusion and differentiation of myoblasts into myotubes was induced at 60% confluence with differentiation medium (DMEM containing 5% horse serum) for 5–8 days.

### Immunocytochemistry

Attached hippocampal neural stem cell-derived neurospheres, lentivirally transduced HEK293FT cells, C2C12 myoblasts or differentiated myotubes were grown on coverslips (thickness 170 µm +/− 0,005 µm), and were fixed using 4% phosphate buffered paraformaldehyde (PFA; pH 7.4) for 20 min at room temperature (RT) followed by 3 wash steps in 1×phosphate buffered saline (PBS) for 5 min. Fixed cells were permeabilized with 0.02%–0.2% Triton X-100 followed by blocking using 5% normal goat or donkey serum (Dianova, Hamburg, Germany) and incubation with primary antibodies for 2 h at RT at the following dilutions: mouse anti-nestin 1∶100 (clone Rat 401, Merck Millipore, Schwalbach, Germany), mouse anti p24 1:100 (clone 7A8.1, Merck Millipore), rabbit anti α-tubulin 1∶200 (Sigma, T9026, DM1A), mouse anti α-actinin 1∶200 (Sigma, A7811, clone EA53). The secondary QD655 labeled IgG F(ab)2 (1∶50) or Alexa700-conjugated antibodies (1∶500) (both Molecular Probes/Life Technologies, Darmstadt, Germany) were incubated for 1 h at RT in PBS with 5% BSA followed by three wash steps with PBS and post-fixation using 4% phosphate buffered PFA (pH 7.4) for 20 min at RT. Actin labelling was performed with Atto488 conjugated phalloidin (0.1 µM, Sigma). Nuclear counterstaining was performed with DAPI (1 µg/ml, Molecular Probes/Life Technologies) followed by mounting using Mowiol (Carl Roth, Karlsruhe, Germany).

### Image Acquisition and Image Analysis

A standard confocal laser scanning microscope (Zeiss LSM710), equipped with 405 nm diode (30 mW), 488 nm argon (20 mW) and 561 nm diode (10 mW) lasers and a 63×NA1.4 Plan Apochromat objective was used for image acquisition. Typical laser intensities applied were 1–5% and 20–40% of the 405 nm laser, 5–10% and 50–70% of the 488 nm argon laser, 10% and 70% of 561 nm laser for QD and QDTX imaging, respectively. This corresponds to 0.09–0.2 mW (405 nm laser), 0.08–0.14 mW (488 nm laser) and 0.38 mW (561 nm laser) of laser power for QDTX, measured in the objective plane of the 63×objective.

For comparability of QD and QDTX images, the same PMT detector was used with identical settings for gain, digital gain and offset with emission filter settings 635–675 nm for QD imaging, 580–610 nm for QDTX imaging. The pinhole aperture was 0.7 airy unit (AU) for QDTX with identical section thickness for both the QD and QDTX channels and additional fluorophores if applicable. Pixel size for image acquisition was ≤60 nm with 12 bit data depth and unidirectional scanning. Z-sectioning was at 0.1–0.2 µm per Z-layer, in order to fulfill Nyquist criteria for further image processing by deconvolution. Scan speed was typically set to 1–2 µsec pixel dwell time with line averaging of 2–4.

Spectral scans of QD655 clusters (1 nM antibody conjugate solution dried on coverslips and mounted with Mowiol) in the range of 550–700 nm were acquired with a standard Leica SP5 confocal microscope with a 63×HCX Pl APO lambda blue NA1.4 objective and a 405 nm diode (30 mW), 488 nm argon (20 mW) or 561 nm DSSP (10 mW) laser. Laser intensities of 10%, 50% and 100% correspond to 0.05 mW, 0.26 mW and 0.58 mW (405 nm laser), 0.085 mW, 0.56 mW and 1.07 mW (488 nm laser), 0.01 mW, 0.039 mW and 0.084 mW (561 nm laser) respectively, measured in the objective plane of the 63×objective. Multicolor imaging of Atto488, QD655, Alexa700 without spectral unmixing was performed by acquiring first Atto488 (Em 500–530 nm) and Alexa700 (Em 700–750 nm) with the 488 nm and 633 nm laser line, respectively, followed by QD and QDTX imaging with 405 nm excitation.

Multicolor imaging of four fluorophores (Atto488, QD655 and Alexa700 including DAPI labeling) required spectral unmixing. First, Atto488 (Em 500–530 nm) and Alexa700 (Em 700–750 nm) were acquired with the 488 nm and 633 nm laser lines, respectively. DAPI, which is at the same time excited by the strong 405 nm laser for QDTX, gives rise to a signal in the QDTX channel. The QDTX signal was subsequently detected by a spectral scan from 430 nm to 610 nm, which enables unmixing of both the DAPI and QDTX signal by post processing of the image with the Zeiss Zen Software.

Deconvolution of QD and QDTX images was performed with the SVI HuygensPro software v. 4.2 (Scientific Volume Imaging B.V., VB Hilversum, Netherlands). Here we used a theoretical PSF assuming 1-photon excitation for the QD images and 2-photon excitation for the QDTX images, letting the classic maximum likelihood estimation (CMLE) algorithm running for at least 40 iterations. Line profiles of viral particles and microtubule were generated with the twin slicer option of this software, in order to measure the FWHM of these structures. All theoretical PSFs were generated with HuygensPro 4.2 PSF distiller assuming 1-photon, 2-photon or 3-photon excitation and the appropriate wavelength for excitation and emission. The theoretical PSFs were used to convolve an example image with the 1-photon, 2-photon and 3-photon functions. First, we imported the Huygens PSFs as a series of text images, and then used the properly scaled middle frame of this Z stack for 2D convolution of the “blobs” example image from ImageJ (NIH, http://rsbweb.nih.gov/ij/).

All other image processing was done with ImageJ (NIH, http://rsbweb.nih.gov/ij/)/FIJI (FIJI project, General Public License, http://www. http://fiji.sc/).

## Results and Discussion

In order to demonstrate the efficient formation of tri-excitonic (TX) states with a commercial confocal microscope and CW lasers of various emission wavelengths we used aggregates of QD655 labeled antibodies immobilized on coverslips (Figure 1B and Figure S1) and acquired spectral emission scans from 550 nm to 700 nm with a bandwidth of 5 nm. Images of QD clusters taken from the spectral scan (Figure 1B, from 595 to 600 nm for TX and 650–655 nm for normal QD imaging with MX) reveal more structural details due to the improved lateral resolution of QDTI. The absolute and relative amount of TX states formed compared to MX states increases with increasing laser power (Figure 1B). The intensity of the 405 nm laser applied during the spectral scans of the TX states in QD antibody clusters was comparable to that applied for QD and QDTX scans of the cell samples. The effect of TX formation is due to the excitation efficiency of QD655 (Figure 1A/B) especially high at 405 nm, but also clearly detectable by the 488 nm and 561 nm laser lines (Figure S1). These results convincingly demonstrate the efficient generation and detection of TX states from QD655 labeled antibodies with various standard CW lasers at commercial confocal laser scanning microscopes, which results in a lateral and axial resolution enhancement.

In order to show the applicability of QDTI as a simple and flexible method to obtain an improved optical resolution of cellular structures, we examined various biological samples (lentiviral microvesicles, microtubules and intermediate filaments), and combined QDTI with image deconvolution.

Deconvolution is an image restoration technique that reduces photon noise and blur generated by the optical system, thereby improving image contrast and resolution [12]. However, for many newly developed imaging techniques, such as QDTI, structured illumination or light-sheet based microscopy, the necessary new deconvolution algorithms are not yet commercially available. The use of an experimental PSF, acquired from an individual QD655 under conditions comparable to QDTI of cellular samples, was limited due to the low light efficiency and detector sensitivity of a confocal microscope. Therefore, we decided to use commercially available software (HuygensPro, SVI) and an iterative algorithm (classic maximum likelihood estimation, CMLE; see [13]) with a theoretical PSF. As the spectral range detected in QDTI (580–610 nm) is likely to be a combination of both BX and TX photons at an unknown ratio, we tested the effect of various deconvolution conditions, i.e. those that assume 2-photon or 3-photon excitation. We calculated the PSFs for 1-photon (conventional QD images, MX) as well as 2-photon (BX) and 3-photon absorption (TX) of QD655 accounting for the correct excitation and emission wavelengths in contrast to standard multi-photon absorption (Figure 1C). The 3D shape of the calculated PSFs for bi- and tri-exciton absorption, as well as the effect of convolution of artificial structures (Figure 1C) by these calculated PSFs show a high degree of similarity between BX and TX compared to MX. This is corroborated by line profiles showing that BX and TX absorption have a similar, although gradually different effect on the separation of closely arranged structures (line profile 1 and 2) and on the revelation of hidden substructures (line profile 1 and 3). Because the deconvolution of QDTX images with the 3-photon CMLE algorithm resulted in deconvolution artifacts in real biological samples (e.g. the appearance of a periodic background pattern, see Fig. S2), we decided to use the 2-photon excitation model for deconvolution of QDTX images but accounting for the wavelength used in QDTI.

**Figure 2:**
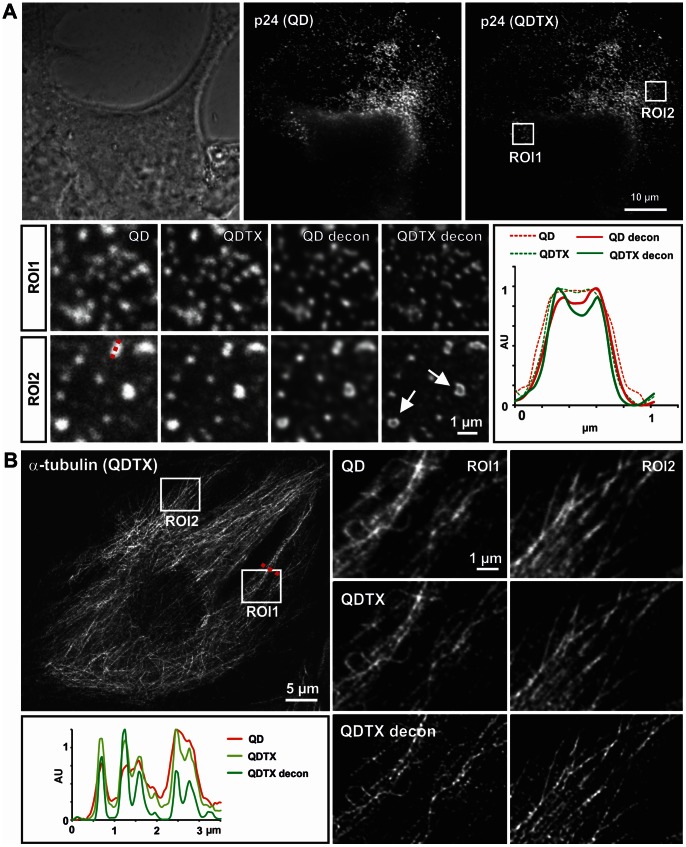
Increased resolution of different cellular structures by QDTI and subsequent image deconvolution. (A) Lentivirally transduced HEK293FT cells were immunocytochemical stained for p24. Phase contrast images demonstrate cellular fusions, typical for lentivirally infected cells (upper panel, left). Imaging of QD655 stained p24 reveals the presence of viral particles at the surface of the infected cells and the release of viral vesicles into the adjacent areas. QDTI with 488 nm excitation provides improved resolution further improved by subsequent deconvolution (compare QD with QDTX and QDTX with QDTX deconv). This effect is especially prominent in larger hollow clusters, which were detected in addition to small microvesicles with p24 localized at the periphery (arrows). (B) Microtubuli (MT) in C2C12 myoblast cells were visualized by immunocytochemical staining using α-tubulin antibody and QD655 coupled secondary antibodies. MT were imaged by conventional QD technique, and with QDTI using 405 nm excitation followed by image deconvolution. Magnifications of ROI1 and ROI2 and line profiles of the indicated area (broken line) show the effect of QDTI and subsequent deconvolution on the resolution of individual microtubuli compared to QD images. Pinhole: 0.7 AU.

In order to visualize the spatial distribution and size of viral microvesicles, we infected HEK293FT cells with a lentivirus. Imaging of cells cultivated for 24 h post-infection clearly revealed typical fusion events of infected HEK cells (Figure 2, upper panel, phase contrast image). Moreover, the QD signal revealed the presence of viral microvesicles at the surface of infected cells (Figure 2, upper panel, QD and QDTX). Although single lentiviral vesicles with a size-range of 0.24–0.36 µm (FWHM, mean size 0.28 µm, n = 13) are visible in QD derived images, QDTX images clearly show an improved resolution compared to conventional QD images, especially after deconvolution. The same sample of HEK cells with p24-labelled virus-microvesicles was scanned with 488 nm (Figure 2) and 561 nm laser lines (data not shown) for QDTI excitation, in order to demonstrate the flexibility and applicability of QDTI for microscopes with different laser configurations. The QDTX images obtained with different excitation wavelength revealed very similar structural details of the vesicles but resulted in slightly different size estimate for single microvesicles. Line profiles of individual particles revealed a size of 0.23 µm (FWHM, mean, n = 13) for QDTI at 488 nm excitation and 0.25 µm (FWHM, mean, n = 13) for QDTI at 561 nm excitation. Deconvolution of QD and QDTX images provided further increase in resolution, thus revealing new structural details (Figure 2A, line profile, diagram). In addition to small microvesicles, we also observed larger, hollow p24-labelled viral microvesicles with a diameter of 0.7 to 0.9 µm (Figure 2A, arrows).

As a well-established biological structure that is often used to assess the optical resolution of a microscopy technique, we also visualized microtubuli (MT) in C2C12 myoblast cells by α-tubulin immunofluorescence staining (Figure 2B). These cells show linear arrays of densely packed MT during early differentiation into myotubes [14] in addition to more radially arranged MT. Acquisition of QD images and QDTI of microtubular structures were performed using a 405 nm laser. As the magnifications indicate, the structural details of MT are more clearly resolved in QDTX images than in images acquired with conventional QD655 imaging. A significant further improvement of resolution was achieved by deconvolution of the QDTX images. Closely arranged individual MT are clearly distinguishable, unlike in the conventional QD images, a finding supported by line profiles displaying the intensity distribution of individual MT in the QD, QDTX and deconvolved QDTX images. The diameter of a typical microtubule is 0.26 µm in the conventional QD image, 0.18 µm in the QDTX image and 0.13 µm after deconvolution of the QDTX image (Figure 2B, line profile, all FWHM). Thus, the achieved resolution enhancement is at least 1.4 fold by QDTI and 1.9 fold by subsequent deconvolution of the QDTX images compared to the conventional QD image.

**Figure 3:**
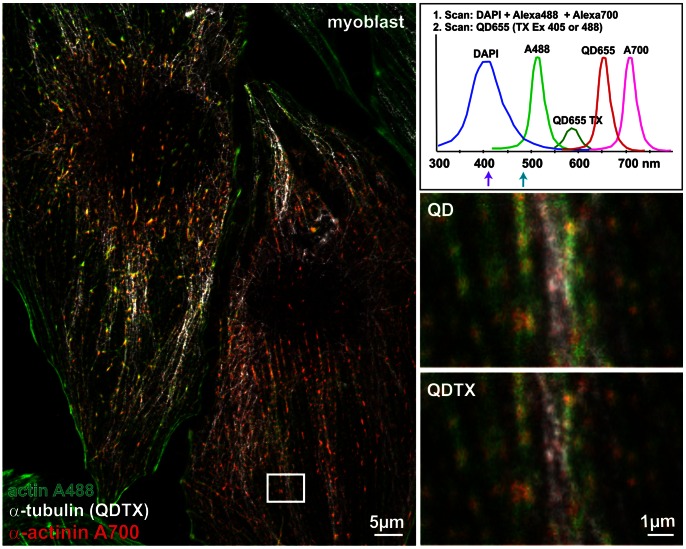
Improved correlation of the localization of cellular structures as revealed by QDTI combined with color multiplexing. Differentiated myoblasts stained for actin filaments (phalloidin Atto488), α-actinin Alexa700 and α-tubulin QD655. QD labeled α-tubulin was acquired by QDTI, α-actinin and actin using conventional confocal microscopy. Magnifications demonstrate the improved structural resolution and localization details of microtubules in the QDTX image in relation to adjacent actin and actinin filaments. The fluorescence spectra of the applied fluorophores that can be used in combination with QD655 labeling and QDTI (top right; spectra redrawn based on Invitrogen data). Pinhole: 0.7 AU. For further imaging conditions, see Table 1.

**Figure 4:**
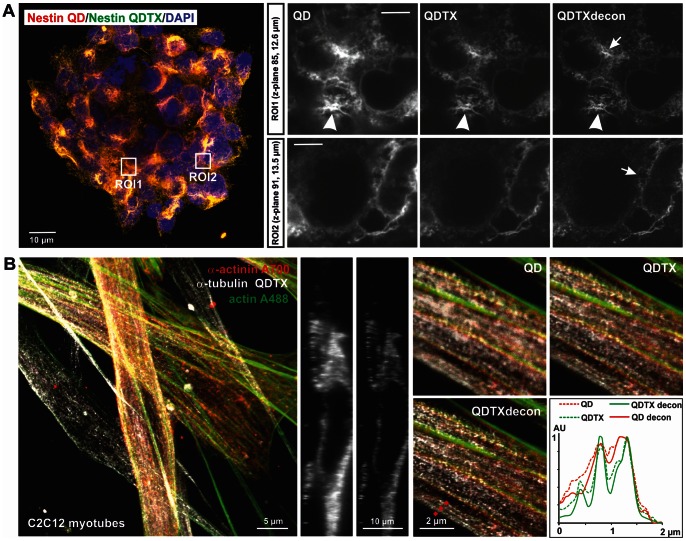
Improved resolution of cellular structures in large and thick biological samples by QDTI, deconvolution and color multiplexing. (A) Hippocampal neural stem cells grown as neurospheres were stained for nestin QD655 and counterstained for DNA using Dapi. QDTI with subsequent deconvolution results in improved resolution, with partially depolymerized nestin filaments that are not resolved by QD (arrows). Arrowheads indicate filamentous nestin. (B) Layers of myotubes formed by fused, differentiated C2C12 myoblasts stained for actin filaments (Atto488), α-actinin Alexa700 and α-tubulin QD655. Orthogonal yz sections show the improved axial resolution and sectioning effect of the QDTX image compared to the normal QD image. Parallel arrays of microtubuli and their localization relative to actin and actinin structures are better resolved in QDTX and QDTXdecon images, even in deeper layers of the sample. Pinhole: 0.7 AU.

QDs exhibit a broad absorption spectrum and a narrow emission spectrum, which is ideal for spectral multiplexing. Moreover, the efficient generation of tri-excitonic states is possible using different excitation wavelength (Figure 1B and Figure S1). We tested various possibilities of color multiplexing to combine the resolution improvement achieved by QDTI of QD655 labeled structures with other cellular structures labeled with commonly used dyes. Limiting factors for potential fluorophore combinations are (i) the cross excitation of dyes during QDTI and bleed-through of these fluorophores into the QDTX channel, and (ii) bleaching of fluorophores due to the higher laser power applied during QDTI. By proper selection of fluorophores, tuning of the excitation wavelength for QDTI and careful adjustment of imaging conditions, QDTI in combination with up to two additional fluorophores is possible (see Table 1). Three-color imaging of QD655 labeled α-tubulin with Atto488 phalloidin (actin) and Alexa700 labeled α-actinin (see Figure 3 and Figure 4B) requires the separate acquisition of Atto488 and Alexa700, followed by QDTI at 405 nm excitation with a narrower band pass filter for QDTX detection.

**Table 1:** Scheme for color multiplexing with possible fluorophore combinations and conditions for image acquisition.

	DAPI		QD655 TX	A700
Scan 1	Ex405/Em415-470			Ex633/Em700-750
Scan 2			Ex488/Em560-610	
		**A488**	**QD655 TX**	**A700**
Scan 1		Ex488/Em500-530		Ex633/Em700-750
Scan 2			Ex405/Em580-610	
	**DAPI**		**QD655 TX**	**A700**
Scan 1	Ex405/Em415-470	Ex488/Em500-530		Ex633/Em700-750
Scan 2			Ex405/Em 430-610 nm (spectral scan)	

QDTI with excitation of 458 nm or 488 nm would result in cross excitation and bleed-through of Atto488 into the QDTX channel. QDTI with QD655 in combination with DAPI as nuclear marker (see Figure 4A) and Alexa700 as potential further dye can be achieved as sequential scan of the two organic fluorophores first, followed by QD655 imaging. QDTI was conducted specifically with 488 nm as excitation wavelength in order to prevent cross excitation of DAPI. Spectral multiplexing of QDTI, with up to two conventional organic fluorophores, works best in two consecutive scans, as this minimizes photo bleaching due to higher laser powers applied during QDTI. The excitation wavelength for QDTI has to be adapted to prevent cross excitation of spectrally similar fluorophores. In four-color multiplexing (QD655, DAPI, Atto488 and Alexa700), cross excitation of one fluorophore cannot be prevented and a spectral emission scan followed by spectral unmixing must be applied.

Numerous microscopy methods with improved optical resolution, developed in the last few years, are limited in their applicability to thin samples or cell monolayers. However, biomedical applications would require imaging methods where the improved optical resolution remains valid for biological samples formed by multiple cellular layers. In these samples, resolution is often severely reduced by spherical aberrations or light scattering effects. Improved axial and lateral resolution is especially beneficial in deeper layers of the sample, where these blurring effects are more pronounced than in single cell layers. We applied QDTI to biological samples of extended axial and lateral dimensions such as multi-cellular neurospheres (Figure 4A) or skeletal myoblasts fused and differentiated to form stacks of myotubes (Figure 4B).

For the imaging of nestin and its differentiation-induced disassembly we cultivated murine neurospheres in the presence of 10% FCS, which usually induces rapid spontaneous differentiation of neural stem cells. Nestin, a stem cell-specific intermediate filament is an important dynamic structure that is rapidly depolymerized after induction of differentiation. QD and QDTX revealed an improved resolution of partially filamentous nestin, demonstrating signs of depolymerization (see Figure 4A, arrows). This improved resolution was especially apparent if deconvolution was applied to the QDTX images (Figure 4A, lower panel). Under certain conditions, skeletal muscle cells are able to fuse to form layers of differentiated, multinucleated myotubes. The differentiation and fusion process is accompanied by a dramatic reorganization of the cellular cytoskeleton [15], [16], which includes the integration of actin and α-actinin into pre-myofibrillar structures (Figure 3), and the rearrangement of radially distributed microtubuli into tightly bundled arrays (Figure 4B). Here, orthogonal sections show the improved axial resolution and sectioning effect of the QDTI compared to confocal QD imaging. Individual microtubuli of tightly bundled arrays demonstrate better resolution in the QDTX image compared to the normal QD image. This effect of enhanced resolution is further improved by deconvolution of the QDTX image, which is especially prominent in deeper layers of the sample (see magnified area). Line profiles from a section 7 µm deep in the sample show the dramatic effect of resolution enhancement on densely arranged arrays of microtubuli by QDTI and further deconvolution of the QDTX image.

Here we have demonstrated the efficient generation of tri-excitonic states from QD655 with various CW laser lines of a standard confocal microscope. In addition, we have shown a clear improvement of structural details seen in QDTX images of QD clusters and of various subcellular structures such as microtubuli, nestin-filaments and lentiviral microvesicles. The application of image restoration by deconvolution further improved the contrast and resolution of the QDTX images. We have successfully applied QDTI to image lentiviral microvesicles, as an example of a biological structure with a typical size near to the diffraction limit of conventional light microscopy. Newly assembled lentiviral virions are usually released from the host cell in a process called “budding”, leading to formation of infectious exosomal microvesicles [17]. Such single microvesicles have a size of approximately 50–250 nm and can fuse and form larger structures [18]. Recent studies using super-resolution microscopy techniques such as STORM or PALM [19] [20]–[24] provided a detailed view on the biology of lentiviruses. In most cases, the sizes of lentiviral virions measure ∼70–100 nm in diameter [19]–[23]. However, all of these approaches require special equipment (for example, pulsed laser sources) to achieve the required higher resolution. The method presented here provides an easy and cost-effective alternative to study lentiviral microvesicles, their release via budding, and infection processes with sub-diffraction resolution. Using a combination of QDTI imaging with subsequent deconvolution, we demonstrated a clearly visible improvement of resolution, allowing imaging of single lentiviral microvesicles with a size of 0.23 µm. Importantly, our method allowed the observation of hollow viral microvesicles, with p24 localized in the periphery only resolved in QDTI with subsequent deconvolution (see Figure 2A, lower panel).

The resolution enhancement effect is especially prominent in samples formed by multiple layers of cells, e.g. fused differentiated muscle cells and stem cells clusters. In samples with extended axial dimensions, resolution is often strongly degraded by blurring effects due to spherical aberrations or light scattering. Such thick samples are not easily adoptable by other improved optical resolution methods. Intermediate filaments represent another type of biological specimen with a size below the diffraction limit. Our method allows imaging of intermediate filaments such as nestin with obviously improved resolution (Fig. 4A, arrowheads), thus providing a powerful tool to study the dynamics of such structures. The combination of QDTI of QD655 with multi-color confocal imaging of standard green and far-red organic fluorophores as well as nuclear stains, substantially extends the applicability of this technique and allows greatly improved visualization of cellular details. In summary, we propose the use of QDTI with QD655 labeled cellular structures, in combination with image deconvolution and color multiplexing, as a simple and flexible method of obtaining images with improved optical resolution. Because this method applies a standard confocal microscope, QDTI, in contrast to other techniques, offers sub-diffraction resolution that is not restricted to small areas of a cell and can be applied to relatively thick specimens formed by multiple layers of cells.

## Supporting Information

Figure S1 **The effect of various excitation wavelengths and laser intensities on the generation of tri-excitonic states demonstrates the flexibility and efficiency of QDTI.** Clusters of QD655 labeled antibodies were excited with 405 nm, 488 nm or 561 nm laser lines of increasing intensities and imaged with a spectral scan between 550 nm and 700 nm. The generation of bi- and tri-excitonic states is most efficient with the 405 nm excitation whilst it is still possible with 488 nm and 561 nm laser lines, in close correspondence with the excitation coefficient of QD655. Laser intensities for this set of experiments correspond to 10%, 50% and 100% of maximum laser power.
(TIF)

Figure S2 **The effect of a PSF with 2-photon or 3-photon absorption assumption on deconvolution of images.** QDTX images of viral particles from Figure 2A were deconvolved with the CMLE algorithm under identical conditions, i.e. number of iterations, background (BG), signal to noise ratio (SNR) but using a theoretical PSF for either the 2-photon or 3-photon absorption case. Deconvolution with the 3-photon CMLE algorithm showed deconvolution artifacts in the cellular background signal (e.g. the appearance of a background pattern) and the structure of the viral particles. Deconvolution with the 2-photon CMLE was in our hands more robust towards biological samples with inhomogeneous SNR and BG levels. In general, when applying deconvolution algorithms, a careful test of the effect of different BG and SNR settings on the structure of the real biological samples is advisable.
(TIF)
