# Supplementary figures and images for: Simple Method for Sub-Diffraction Resolution Imaging of Cellular Structures on Standard Confocal Microscopes by Three-Photon Absorption of Quantum Dots
Source: PLoS One. 2013 May 21;8(5):e64023. doi: 10.1371/journal.pone.0064023 (PMC3660314; doi:10.1371/journal.pone.0064023)

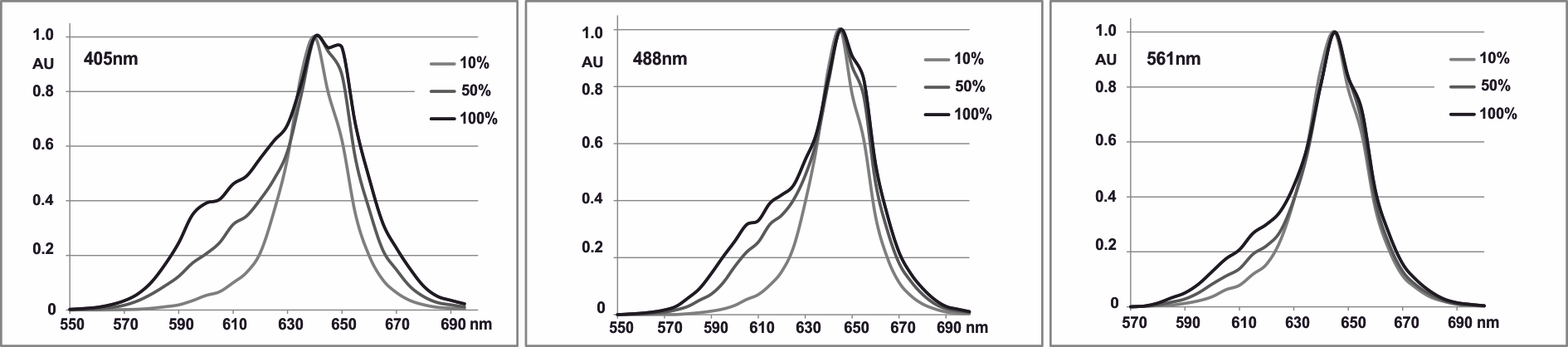

Supplement: Figure S1 — The effect of various excitation wavelengths and laser intensities on the generation of tri-excitonic states demonstrates the flexibility and efficiency of QDTI. Clusters of QD655 labeled antibodies were excited with 405 nm, 488 nm or 561 nm laser lines of increasing intensities and imaged with a spectral scan between 550 nm and 700 nm. The generation of bi- and tri-excitonic states is most efficient with the 405 nm excitation whilst it is still possible with 488 nm and 561 nm laser lines, in close correspondence with the excitation coefficient of QD655. Laser intensities for this set of experiments correspond to 10%, 50% and 100% of maximum laser power. (TIF) [file pone.0064023.s001.tif]

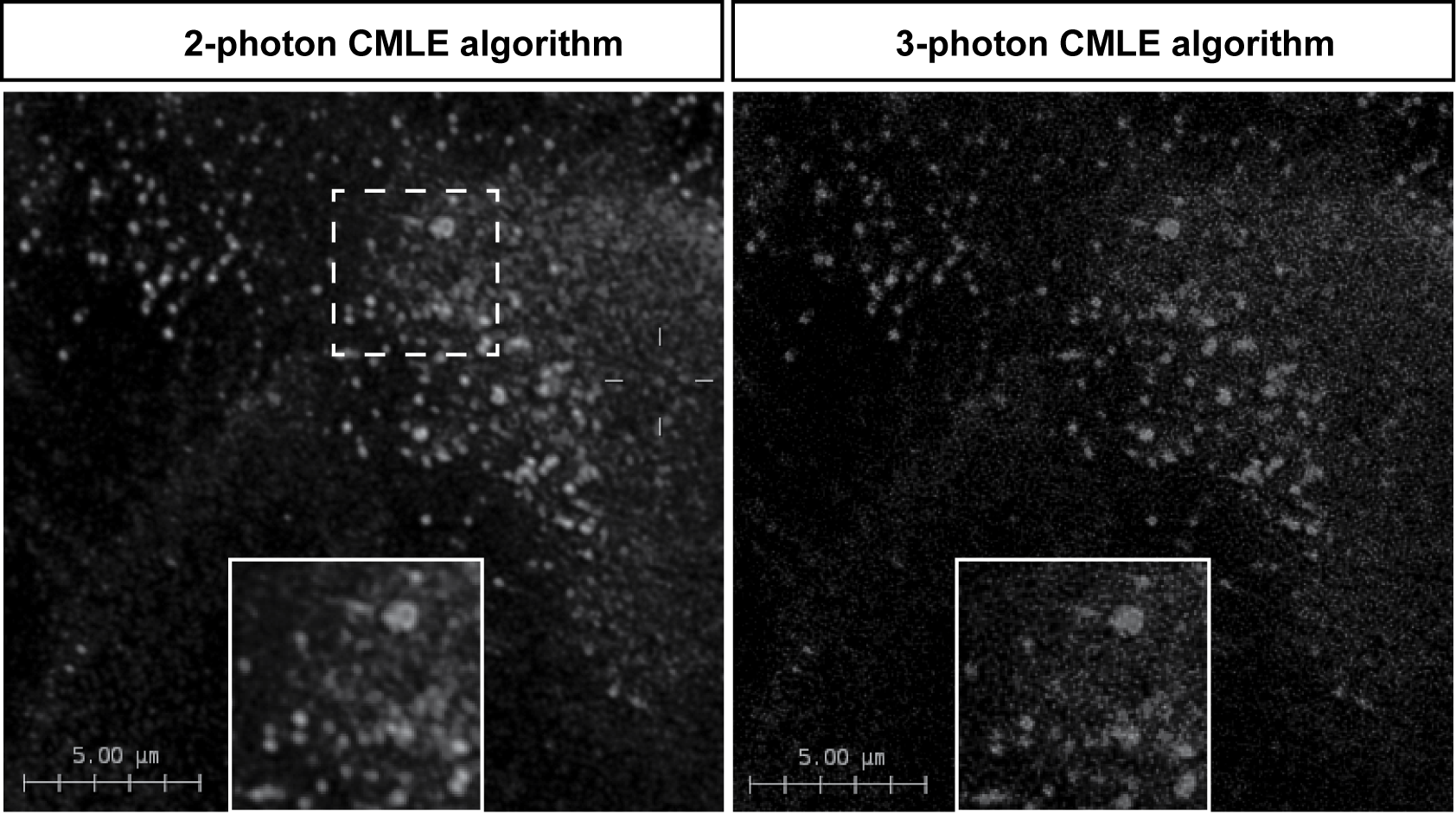

Supplement: Figure S2 — The effect of a PSF with 2-photon or 3-photon absorption assumption on deconvolution of images. QDTX images of viral particles from Figure 2A were deconvolved with the CMLE algorithm under identical conditions, i.e. number of iterations, background (BG), signal to noise ratio (SNR) but using a theoretical PSF for either the 2-photon or 3-photon absorption case. Deconvolution with the 3-photon CMLE algorithm showed deconvolution artifacts in the cellular background signal (e.g. the appearance of a background pattern) and the structure of the viral particles. Deconvolution with the 2-photon CMLE was in our hands more robust towards biological samples with inhomogeneous SNR and BG levels. In general, when applying deconvolution algorithms, a careful test of the effect of different BG and SNR settings on the structure of the real biological samples is advisable. (TIF) [file pone.0064023.s002.tif]
